# High First Trimester Levels of TSH as an Independent Risk Factor for Gestational Diabetes Mellitus: A Retrospective Cohort Study

**DOI:** 10.3390/jcm11133776

**Published:** 2022-06-29

**Authors:** Juan Jesús Fernández Alba, María Castillo Lara, José Manuel Jiménez Heras, Rocío Moreno Cortés, Carmen González Macías, Ángel Vilar Sánchez, Florentino Carral San Laureano, Luis Javier Moreno Corral

**Affiliations:** 1 Department of Obstetrics and Gynecology, University Hospital of Puerto Real, Ctra. Nacional IV, Km 665, 11510 Puerto Real, Spain; mariacastillo.747@gmail.com (M.C.L.); rocio.mc.91@gmail.com (R.M.C.); carmengonzalezmacias.1973@gmail.com (C.G.M.); angel.vilar.sspa@juntadeandalucia.es (Á.V.S.); 2 Institute of Research and Innovation in Biomedical Sciences of the Province of Cadiz (INiBICA), Hospital Universitario Puerta del Mar, Avda. Ana de Viya, 21, 11009 Cádiz, Spain; 3 Department of Endocrinology and Nutrition, University Hospital of Puerto Real, Ctra. Nacional IV, Km 665, 11510 Puerto Real, Spain; florencarral@hotmail.com; 4 Department of Nursing and Physiotherapy, University of Cádiz (UCA), Avda. Ana de Viya, 52, 11009 Cádiz, Spain; luis.moreno@uca.es

**Keywords:** gestational diabetes mellitus, subclinical hypothyroidism, subclinical hyperthyroidism, thyroid stimulating hormone, thyrotropin, thyroid antibodies

## Abstract

Although numerous articles have found an association between alterations in thyroid function and the risk of gestational diabetes mellitus (GDM), other studies have failed to demonstrate this association. This may be due to the different cut-off points used to define subclinical hypothyroidism. We aim to clarify the role of thyroid stimulating hormone (TSH) level in GDM within pregnant women with normal free thyroxine (fT4) levels. This retrospective cohort study was performed in 6775 pregnant women. The association between TSH and GDM was assessed by bivariate and multivariate logistic regression. Pregnant women with subclinical hypothyroidism are at significantly greater risk for GDM when compared with euthyroid pregnant women (OR = 1.85; 95% CI = 1.36–2.52). We have also observed that TSH levels increase the risk of GDM within euthyroid pregnant women, since the TSH levels between 2.5 and 4.71 showed a higher risk of GDM than those whose TSH levels are between 0.31 and 2.49 (OR = 1.54; 95% CI = 1.28–1.84). In addition, pregnant women with positive thyroid antibodies have almost 2.5 times the risk of developing GDM (OR = 2.47; 95% CI = 1.57–3.89). Our results support that in pregnant women with normal fT4 levels, higher first trimester TSH level implies a higher risk of GDM.

## 1. Introduction

Gestational diabetes mellitus (GDM) is defined as glucose intolerance that is diagnosed for the first-time during pregnancy, but usually resolves soon after delivery [1].

GDM is associated with adverse perinatal outcomes, including macrosomia, shoulder dystocia, caesarean delivery, preeclampsia, and neonatal hypoglycemia [2,3]. Moreover, GDM is a marker for future health problems to both the mother and her child, including obesity, type 2 diabetes, metabolic syndrome, and cardiovascular disease [4].

Both clinical and subclinical hypothyroidism (SCH) are insulin-resistant states [5]. Given that a progressive increase in insulin resistance also occurs during pregnancy, it seems reasonable to think that pregnant women with hypothyroidism would be exposed to a greater risk of suffering from GDM.

Although numerous articles have found an association between alterations in thyroid function and the risk of GDM [6,7], other studies have failed to demonstrate this association [8]. Most studies focus on the relationship of SCH with GDM. Given that the definition of SCH is based on the application of a specific cut-off point for TSH, it is logical that the results vary between authors depending on the cut-off point applied. In fact, until 2017, the ATA recommended considering SCH when, with normal fT4, the woman presented TSH greater than 2.5 mIU/L [9]. However, since 2017, this cut-off point has been set at 4 mIU/L [10]. A recent meta-analysis [11] shows that the differences in risk observed between some studies and others could be due, at least in part, to the different cut-off points applied.

Our study aims to clarify whether, in women with normal fT4 levels, TSH constitutes a risk factor for GDM regardless of the cut-off point applied to diagnose SCH. In addition, we intend to determine how the presence or absence of antithyroid antibodies influences the risk of developing GDM.

## 2. Materials and Methods

This retrospective cohort study was conducted at the University Hospital of Puerto Real (Spain) between January 2014 and January 2021. The study was performed in accordance with the Declaration of Helsinki and was approved by the Biomedical Research Ethics Committee of the province of Cádiz (SPAIN) with the protocol number 02 of 31 July 2020.

All pregnant women who underwent routine prenatal care including screening for subclinical hypothyroidism in the first trimester were potentially eligible for the study.

Women with a pre-pregnancy diagnosis of diabetes mellitus, hyperthyroidism or hypothyroidism were excluded from the study.

During the first trimester routine visit information about maternal age, initial weight and height, gravidity, parity, prior or current pregnancy complications and BMI was collected by interview and physical examination of all participants. Participants were also asked about family history of type 2 diabetes mellitus.

In accordance with the recommendations of the Spanish Society of Obstetrics and Gynecology, in our center, we perform universal screening for SCH during the first antenatal visit, performed between weeks 6 and 11 [12]. A maternal blood sample was collected from all participants at the first antenatal visit and was centrifuged (10 min with rethawing cycles at 3000 rpm) to obtained serum. Serum TSH, free T4 (fT4), anti-Thyroglobulin antibodies (Tg Ab) and thyroid peroxidase antibodies (TPO Ab) were measured by automated electrochemiluminiscent immunoassays (ECLIA) (COBAS^®^. Roche Diagnostics GmbH, Sandhofer Strasse 116, D-68305, Mannheim, Germany).

### 2.1. Definitions

#### 2.1.1. Thyroid Function

For the purposes of this study, we consider normal TSH and free T4 (fT4) values in the first trimester of pregnancy between our own 2.5th percentile and 97.5th percentile.

##### Reference Ranges

1. All pregnant women with a TSH value > 10 mIU/L were excluded from the study because we considered that with this TSH level, it was overt hypothyroidism, regardless of the fT4 values.
2. Normal fT4 level ranges from 0.84 ng/dL to 1.65 ng/dL. All women with fT4 values outside the above range were excluded from the study.
3. Normal TSH level ranges from 0.13 mIU/L to 4.72 mIU/L.

Pregnant women with normal fT4 levels and TSH levels <2.5th percentile were classified as having subclinical hyperthyroidism. Pregnant women with normal fT4 levels and a TSH > 97.5th percentile were considered to have subclinical hypothyroidism.

Patients with first trimester TSH levels between the 2.5th centile and the 97.5th centile were considered euthyroid.

In addition, to clarify whether TSH is associated with a modification of the risk of GDM in euthyroid women, this group was divided into two: a group of pregnant women with TSH between 0.13 and 2.49 mIU/L; and another group of pregnant women with TSH levels between 2.5 and 4.71 mIU/L. We added a cut-off point at 2.5 mIU/L because previously SCH was defined as an elevated TSH > 2.5 mIU/L, as suggested by the American Thyroid Association (ATA) guidelines in 2011. Even though in 2017 the ATA modified the cut-off point for SCH in the case of not having own population curves by setting it at 4 mIU/L, some authors still advocate the diagnose and treatment above these TSH value.

##### Thyroid Antibodies

TPO antibodies (TPOAb) ≥ 34 IU/L were considered positive. Thyroglobulin antibodies (TgAb) ≥ 115 IU/L were considered positive.

#### 2.1.2. Screening and Diagnosis of GDM

A universal screening for GDM with a 50-g oral load was performed at a gestational age of 24 to 26 weeks. It was also performed in the first visit to those pregnant women who present any of the following risk factors: history of familial diabetes (in first-degree relatives), obesity (body mass index (BMI) > 30 kg/m^2^), history of impaired glucose tolerance or GDM, unfavorable obstetric history (repeated miscarriages, fetal death without cause, fetal macrosomia [>4 kg], malformations, or other obstetric or perinatal data suggestive of diabetes) and belonging to an ethnic group with high prevalence.

For the diagnosis of GDM, we follow the criteria of the Spanish Group on Diabetes and Pregnancy (GEDE) [13]. Therefore, the diagnosis of GDM was established when at least two of the following four plasma glucose levels (measured at fasting, 1 h, 2 h, and 3 h after a 100-g oral glucose tolerance test) were equal to or greater than 105 mg/dL, 190 mg/dL, 165 mg/dL and 145 mg/dL, respectively, according to the National Diabetes Data Group and the 3rd Workshop—Conference on Gestational Diabetes Mellitus [13,14].

### 2.2. Statistical Analysis

Categorical data were summarized as count and percentages. The distribution of quantitative data was assessed using the Kolmogorov-Smirnov test and histograms. The two continuous variables included in the study (maternal age and first trimester TSH level) presented a non-normal distribution, so we used the median and the interquartile range to describe them.

Differences between GDM and non GDM groups were studied. Categorical variables were contrasted using the chi-squared test. Continuous variables were contrasted using the Mann-Whitney U test.

#### 2.2.1. Bivariate Analysis

The potential bivariate association between the independent variables and the development of GDM was evaluated by simple logistic regression.

#### 2.2.2. Multivariate Analysis

To assess the independent role of first trimester TSH in development of GDM we estimated two multivariate logistic regression models.

Model 1 included first trimester TSH level as a continuous variable. Due to the distribution of this variable, a log10 transformation of it was included in the model.

Model 2 included TSH as a polytomous variable with the next categories:

- <0.131 mIU/L (subclinical hyperthyroidism)
- 0.131–2.49 mIU/L (Euthyroid 1)
- 2.5–4.71 mIU/L (Euthyroid 2)
- ≥4.72 mIU/L (subclinical hypothyroidism)

As potential confounders, we included initially all the variables with significant association in bivariate analysis. Then, by backward method, we excluded those variables that lost their statistical significance in the multivariate analysis.

A *p*-value less than 0.05 was deemed statistically significant.

For the statistical analysis of the data, we used the software R version 3.6.3 (R Core Team, Vienna, Austria) [15].

## 3. Results

### 3.1. Clinical and Demographic Characteristics

**Figure 1:**
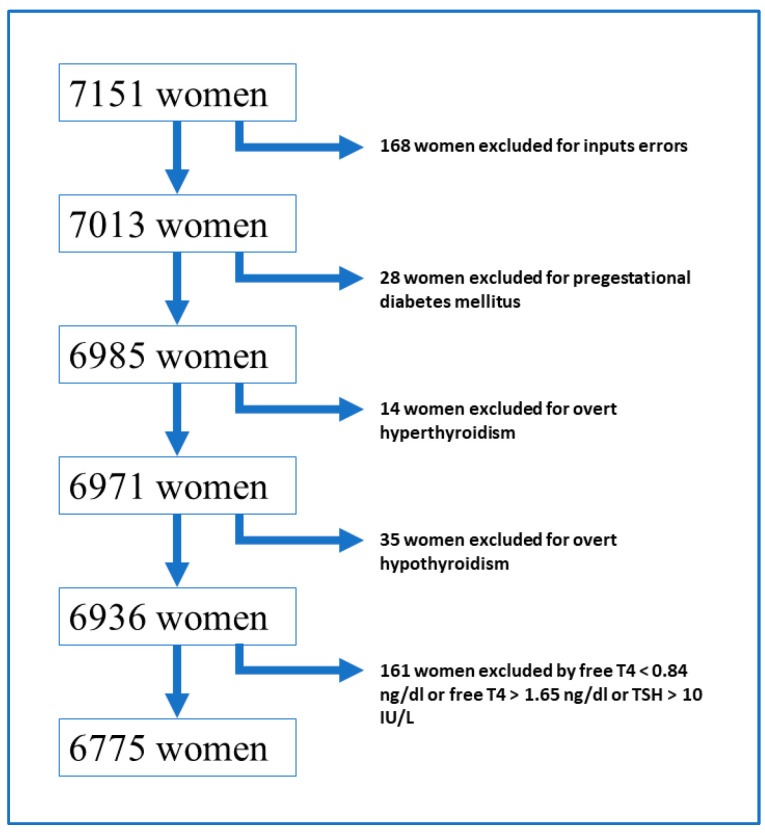
Selection algorithm for the study population; T4 = Thyroxine, TSH = Thyroid Stimulating Hormone.

After applying the exclusion criteria, a total of 6775 Mediterranean pregnant women were included in the study (Figure 1).

Table 1 shows the clinical and demographic characteristics of the whole sample studied, as well as of the pregnant women with GDM and normal glucose tolerance.

When compared with non-GDM women, patients with GDM were significantly older (34.39 years vs. 32.68 years, *p* < 0.001). In addition, the BMI at the beginning of pregnancy was higher in pregnant women with GDM (26.15 kg/m^2^ vs. 24.10 kg/m^2^, *p* < 0.001). In the group of women with GDM, the prevalence of overweight and obesity were much higher than in the group of pregnant women without GDM (33.5% vs. 26.3% and 27% vs. 15.8% respectively, *p* < 0.001). The prevalence of chronic hypertension was significantly higher in the group of women with GDM (3.5% vs. 0.7%).

TSH levels were higher in women with GDM than in women without GDM (2.13 vs. 1.86). Moreover, the proportion of pregnant women with subclinical hyperthyroidism was slightly higher in the group of women with GDM (1.74 vs. 1.59). However, the proportion of pregnant women with subclinical hypothyroidism was significantly higher in the group of women with GDM (8.7% vs. 5.31%).

### 3.2. Bivariate Analysis

After bivariate analysis (simple logistic regression), all the variables included in the study showed significant association with GDM except parity (OR: 1.12; 95% CI 0.96–1.32) (Table 2).

### 3.3. Multivariate Analysis

#### 3.3.1. Model 1

Table 3 shows a summary of the multivariate logistic regression analysis including first trimester TSH level as a continuous quantitative variable (Model 1). As mentioned, due to its distribution, TSH was included in the model as log10 transformation. After analysis, gravidity and recurrent abortion were removed from the model due to lack of statistical significance.

In the same way, after adjusting for the other variables, anti-TPO antibodies also lost their statistical significance, so we decided to incorporate a unified variable that included those women with any positive antithyroid antibodies (anti-TPO and/or anti-TG).

There was no indication of a lack of fit for this model as indicated by the Hosmer-Lemeshow test (*p* = 0.422).

**Figure 2:**
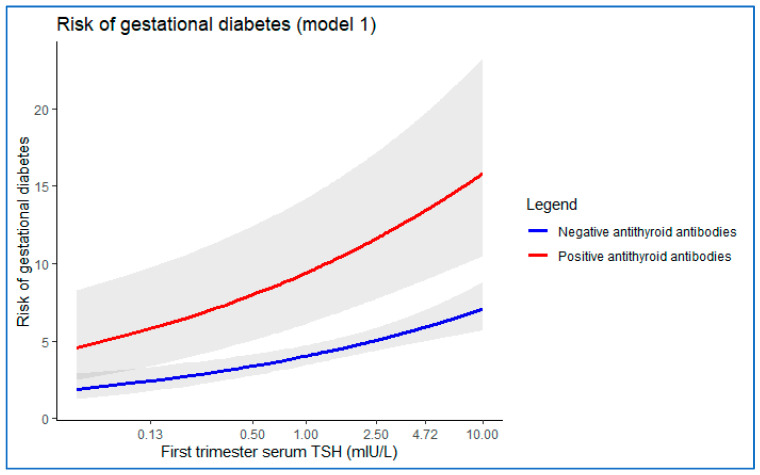
Risk of gestational diabetes mellitus predicted by model 1 for a representative 31-year-old patient, with no family history of type 2 diabetes mellitus, BMI between 18.5 and 24.99 kg/m^2^, and without chronic hypertension.

To illustrate the values predicted by model 1, we calculate the risk of GDM based on the first trimester TSH level of a medium pregnant women, 31 years old, with no family history of type 2 DM, normal BMI at the beginning of gestation, singleton pregnancy and without chronic hypertension. Figure 2 shows the result of applying Model 1 to this typical patient, both with positive and negative anti-TPO antibodies. In this graph, we can see how the higher the TSH level, the greater the risk of GDM, and how women with positive antithyroid antibodies had a 2.47 times higher risk of developing gestational diabetes than women with negative antithyroid antibodies (adjusted OR = 2.38; 95% CI = 1.56–3.89).

#### 3.3.2. Model 2

To clarify the risk associated with different first trimester TSH cut-off points, we repeated the multivariate logistic regression analysis but including TSH levels segmented into four categories described above.

Table 4 shows a summary of this model (Model 2). Again, gravidity and recurrent abortion were removed from the model due to lack of statistical significance, and again there was no indication of a lack of fit for this second model as indicated by the Hosmer-Lemeshow test (*p* = 0.111).

**Figure 3:**
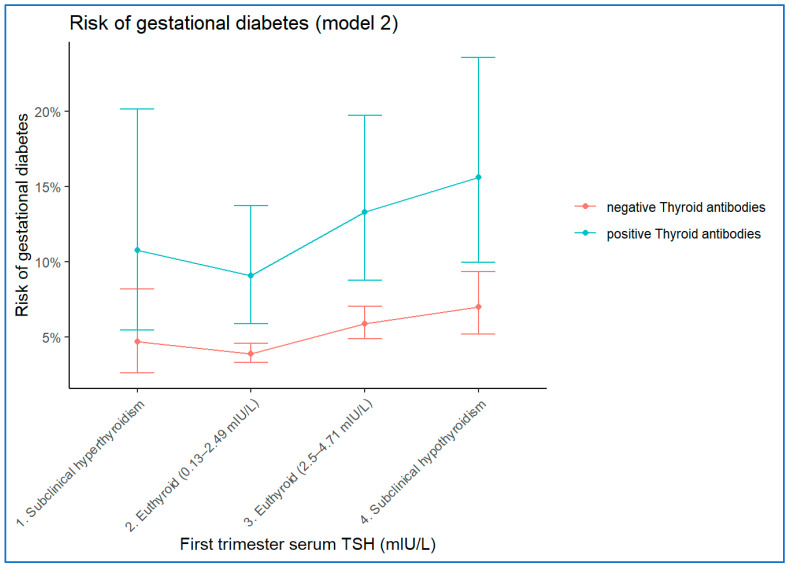
Risk of gestational diabetes mellitus predicted by model 2 for a representative 31-year-old patient, with no family history of type 1 diabetes mellitus, BMI between 18.5 and 24.99 kg/m^2^ and without chronic hypertension.

Compared with euthyroid women with TSH between 0.13–2.5 mIU/L, the women with TSH levels between 2.5 mIU/L and 4.70 mIU/L showed 1.54 times higher risk of GDM (OR = 1.54; 95% CI = 1.28–1.84); and the women with TSH levels equal to or greater than 4.71 mIU/L showed 1.85 times higher risk of GDM (adjusted OR = 1.94; 95% CI = 1.36–2.52). Women with subclinical hyperthyroidism also showed an increased risk of GDM but without statistical significance (OR = 1.21; 95% CI 0.67–2.19). The risk associated with positive antithyroid antibodies estimates by this second model was 2.45 (OR 2.45; 95% CI 1.56–3.86), very similar to the one estimate by Model 1. Figure 3 shows the risk estimated by model 2 for the same typical woman described in Figure 2.

Other risk factors associated with GDM in both models were maternal age, family history of type 2 DM, underweight, overweight, obesity and chronic hypertension (Table 3 and Table 4).

## 4. Discussion

Our results shows that a direct correlation exists between increasing first trimester serum TSH levels and the risk of gestational diabetes. This association remained significant after adjustment for potential confounders, including age, BMI at the beginning of the gestation, family history of type 2 DM, fetus number and chronic hypertension.

Additionally, ours results support that pregnant woman with SCH are at significantly greater risk for GDM when compared with euthyroid women (OR = 1.85; 95% CI = 1.36–2.52). Moreover, within the group of euthyroid women, those with TSH levels between 2.5 and 4.71 also are at higher risk of GDM than those whose TSH levels are between 0.31 and 2.49 (OR = 1.54; 95% CI = 1.28–1.84)

On the other hand, women with positive antithyroid antibodies have almost 2.5 times more risk of gestational diabetes (Model 1: OR 2.47; 95% CI 1.57–3.89) (Model 2: OR 2.45; 95% CI 1.56–3.86).

Many authors have found an association between SCH and the risk of developing GDM with variable results depending on diagnostic cut-off for TSH level, GDM diagnostic criteria, geographic location, ethnicity, micronutrient intake and trimester of pregnancy evaluated [11].

A recent meta-analysis published by Luo et al. [6], including 19 epidemiological studies, concludes that pregnant women with subclinical hypothyroidism had a 1.5-fold increased risk of GDM (OR 1.54; 95% CI 1.03–2.30), very close to the 1.85-fold found in our study.

These results corroborate those previously published by Toulis et al. [7], who performed a meta-analysis including six cohort studies, finding an increased risk of GDM of 1.39 (95% CI 1.07–1.79) in pregnant women with SCH.

Conversely, Maraka et al. [8], in a meta-analysis including 8 studies, found no statistically significant association between SCH and GDM (OR 1.28; 95% CI 0.90–1.81).

The discrepancy in the results could be explained, at least in part, by the different cutoff points used to define the SCH.

In fact, another recent meta-analysis published in 2021 by Kent et al. [11] investigates whether the association between SCH and GDM depends on the cut-off points used. The authors conclude that, regardless of gestational age and antithyroid antibody status, pregnant women with a TSH > 4 mIU/L have 1.60-fold increased odds of GDM (OR = 1.6; 95% CI 1.33–1.93). Only one of the studies included in this meta-analysis explores the risk of GDM in pregnant women with TSH > 4 mIU/L and TA+, concluding that these patients have 3.25-fold increased odds of GDM adjusting by age, maternal pre-gestational BMI, gravidity and parity (OR = 3.25; 95% CI 2.51–4.21) [16].

In the first regression model estimated in our study, in which we include TSH as a continuous quantitative variable, we see that the risk of GDM increases as the TSH value increases. This occurs even in the range that is usually considered normal.

In 2017, the ATA modified the cut-off point for SCH in pregnant women in the case of not having its own population curves, establishing it at 4 mIU/L of first trimester TSH level [10]. However, we find it interesting to note that, in our study, the group of patients with TSH levels in the first trimester between 2.5 mIU/L and 3.99 mIU/L had a 1.5-fold higher risk of developing GDM (adjusted OR 1.54; CI 95% 1.28–1.84) than those women whose TSH was between 0.37 mIU/L and 2.49 IU/L. Our results are consistent with what was published in the meta-analysis by Kent et al. [11], which found an increased risk of GDM in women with SCH based on a cut-off point of less than 4 mIU/L of 1.12 (95% CI 0.94–1.34) in women with AT negative and 2.04 (95% CI 1.32–3.13) in women with AT positive.

The pathophysiological links between SCH and GDM have not been fully elucidated.

The SCH can be considered as an insulin-resistant state predisposing to higher glucose and insulin levels [17,18].

In the meta-analysis published by Toulis et al. [7], the authors list multiple possible mechanisms related to this insulin-resistant state, including:

- Increased levels of free fatty acids [17];
- Impaired ability of insulin to increase blood flow rate to insulin-sensitive tissues [19];
- Abnormal translocation of glucose transporter 2 (GLUT2) resulting in decreased insulin-stimulated glucose transport rate [5];
- Decreased selenium levels [20];

Another mechanism that has been hypothesized as a possible pathophysiological link between SCH and GDM is the increase in oxidative stress associated with hypothyroidism [21]. The release of some inflammatory factors induced by the increase of TSH is proposed by Luo et al. [6] as a mechanism strengthening the process of oxygen stress.

This last hypothesis could explain the fact that, in our study, including only pregnant women with normal fT4 levels, pregnant women with more TSH have a higher risk of GDM, which suggests that the link between TSH and GDM could be determined by extra-thyroid effects. An excellent review on thyroid dysfunction and the risk of diabetes mellitus in general and on the role of TSH in particular can be found in Biondi et al. [22].

Finally, Kent et al. [11], in their meta-analysis, point out that metabolic pathways known to be dysregulated in GDM share a common downstream target of the TSHR, which activates cyclic adenosine monophosphate (cAMP), and, for its part, cAMP would also be involved in the altered endocrine function of syncytiotrophoblasts within the placenta.

In our sample, pregnant women with positive thyroid antibodies showed a 2.5-fold increased risk of GDM. A meta-analysis including cohort and case-control studies found a significant but not strong association between thyroid antibodies and the risk of GDM (RR 1.12; 95% CI 1.03–1.22). However, the same meta-analysis, but including only studies conducted in euthyroid women, did not find a significant association between thyroid antibodies and the risk of GDM (RR: 1.08; 95% CI: 0.97–1.22). The mechanisms involved in the association between thyroid antibodies and GDM has not been fully clarified [23]. The potential link between thyroid antibodies and GDM could be insulin resistance secondary to the action of inflammatory cytokines increased in patients with thyroid autoimmunity [24].

One of the main strengths of our study consists in having treated TSH as a continuous variable in Model 1, which has allowed us to establish an association between the TSH level and the risk of GDM regardless of the cut-off point used to consider SCH.

Our study is not without limitations. The first limitation stems from the retrospective nature of the study. On the other hand, we have not been able to analyze the possible effect of TSH normalization after treatment with levothyroxine in patients with SCH since this information does not appear in our records. Another limitation is not having performed universal screening for dysglycemia during the first antenatal visit. Such data could have helped to understand whether high normal glucose levels themselves are a better predictor of GDM. Moreover, we have not distinguished between early onset GDM and late onset GDM. Further studies including this aspect could help to elucidate whether the risk associated with the increase in TSH levels varies depending on the gestational age at which GDM begins. Finally, having used the NDDG diagnostic criteria, our results could not be extrapolated to populations in which other diagnostic criteria are used.

## 5. Conclusions

In pregnant women with normal fT4 levels, the higher the first trimester TSH level the higher the risk of GDM. Additionally, pregnant women with positive thyroid antibodies have almost 2.5 times the risk of developing GDM than those with negative thyroid antibodies.

## Figures and Tables

**Table 1:** Demographic and clinical characteristics of enrolled pregnant women.

	Total (N = 6775)		GDM (N = 690, 10.2%)		Non GDM (N = 6085, 89.8%)		*p*
	Number (%)	Median (IQR)	Number (%)	Median (IQR)	Number (%)	Median (IQR)	
**Age (years)**		32.87 (7.27)		34.39 (6.96)		32.68 (7.32)	<0.001
**Maternal BMI (kg/m^2^)**		24.33 (6.21)		26.15 (7.76)		24.10 (6.05)	<0.001 <0.001
**Underweight (BMI < 18.5)**	177 (2.6)		18 (2.6)		159 (2.6)		
**Normal weight (BMI between 18.5–24.9)**	3620 (53.4)		255 (37)		3365 (55.3)		
**Overweight (BMI between 25–29.9)**	1833 (27.1)		231 (33.5)		1602 (26.3)		
**Obesity (BMI ≥ 30)**	1145 (16.9)		186 (27)		959 (15.8)		
**Family history of type 2 diabetes mellitus**	1146 (16.9)		231 (33.5)		915 (15)		<0.001
**Gravidity**							0.264
**1**	2699 (39.8)		243 (35.2)		2456 (40.4)		
**>1**	4076 (60.2)		447 (64.8)		3629 (59.6)		
**Parity**							0.752
**Primiparous**	2721 (59.2)		399 (57.8)		3691 (60.7)		
**Multiparous**	1878 (40.8)		291 (42.2)		2394 (39.3)		
**Recurrent abortion**	409 (6%)		63 (9.1%)		346 (5.6%)		<0.001
**Chronic hypertension**	65(1%)		24(3.5%)		41 (0.7%)		<0.001
**Fetus number**							<0.001
**Singleton**	6511 (96.1)		642 (93)		5869 (96.5)		
**Multiple**	264 (3.9)		48 (7)		216 (3.5)		
**Gestational week at delivery**							<0.001
**Preterm birth**	468 (6.9)		66 (9.6)		402 (6.6)		
**Full-term birth**	6307 (93.1)		624 (90.4)		5683 (93.4)		
**Delivery mode**							<0.001
**Vaginal**	5144 (75.9)		474 (68.7)		4670 (76.7)		
**Caesarean section**	1631 (24.1)		216 (31.3)		1415 (23.3)		
**Newborn weight (g)**		3280 (620)		3290 (630)		3280 (610)	0.330
**TSH (mIU/L)**		1.89 (1.63)		2.13 (1.99)		1.86 (1.61)	<0.001 <0.001
**Subclinical hyperthyroidism**	109 (1.61)		12 (1.74)		97 (1.59)		
**Euthyroid**							
**TSH between 0.13–2.49**	4501 (66.44)		399 (57.82)		4102 (67.41)		
**TSH between 2.5–4.71**	1782 (26.30)		219 (31.74)		1563 (25.69)		
**Subclinical hypothyroidism**	383 (5.65)		60 (8.7)		323 (5.31)		
**Thyroid-antibody positive (IU/L)** **(anti-TG > 115 and/or anti-TPO > 34)**	383 (5.65)		126 (18.26)		257 (4.22)		<0.001

IQR = Interquartile range; BMI = body mass index; TSH = thyroid stimulating hormone; anti-TG = anti-thyroglobulin antibodies; anti-TPO = thyroid peroxidase antibodies.

**Table 2:** Risk of gestational diabetes mellitus (bivariate analysis).

	Unadjusted OR	95%CI	*p* Value
**Age (Years)**	1.088	1.071–1.105	<0.001
**Maternal BMI (kg/m^2^)**	1.069	1.056–1.083	<0.001
**Underweight (BMI < 18.5)**	1.494	0.903–2.472	0.121
**Overweight (BMI between 25–29.9)**	1.903	1.577–2.296	<0.001
**Obesity (BMI ≥ 30)**	2.559	2.091–3.133	<0.001
**Family history of type 2 diabetes mellitus**	2.844	2.392–3.381	<0.001
**Gravidity (>1)**	1.245	1.056–1.467	<0.05
**Parity (multiparous)**	1.124	0.959–1.319	0.189
**Recurrent abortion**	1.668	1.260–2.209	<0.001
**Chronic hypertension**	5.317	3.193–8.855	<0.001
**Fetus number (Multiple)**	2.031	1.470–2.807	<0.001
**TSH level at first trimester of gestation (mIU/L)**	1.150	1.093–1.210	<0.001
**Subclinical hyperthyroidism**	1.272	0.692–2.337	0.536
**TSH between 2.5–4.71**	1.440	1.209–1.716	<0.001
**Subclinical hypothyroidism**	1.910	1.423–2.563	<0.001
**Anti-TPO antibodies > 34 IU/L**	2.146	1.275–3.612	<0.01
**Anti-TG antibodies > 115 IU/L**	3.108	1.808–5.343	<0.001
**Thyroid-antibodies positive**	2.568	1.662–3.967	<0.001
**(anti-TG >115 IU/L and/or anti-TPO > 34 IU/L)**			

OR = odds ratio; CI = confidence interval; BMI = body mass index; TSH = thyroid stimulating hormone; anti-TG = anti-thyroglobulin antibodies; Anti-TPO = thyroid peroxidase Antibodies.

**Table 3:** Multivariate logistic regression analysis including first trimester TSH as a continuous quantitative variable (Model 1).

	B	SE	Wald	df	Sig.	Adjusted OR	95% CI by Adjusted OR	
							Lower	Upper
**Age (years)**	0.079	0.008	92.686	1	<0.001	1.083	1.065	1.100
**Family history of type 2 DM**	0.930	0.091	105.125	1	<0.001	2.533	2.121	3.026
**Normal BMI (18.5–24.9 kg/m^2^)**			63.030	3	<0.001			
**Underweight (BMI < 18.5 kg/m^2^)**	0.601	0.265	5.149	1	0.023	1.825	1.085	3.068
**Overweight (BMI 25–29.9 kg/m^2^)**	0.584	0.098	35.564	1	<0.001	1.793	1.480	2.172
**Obesity (BMI ≥ 30 kg/m^2^)**	0.785	0.109	52.103	1	<0.001	2.193	1.772	2.714
**Chronic hypertension**	1.220	0.276	19.562	1	<0.001	3.388	1.973	5.818
**Fetus number (multiple)**	0.669	0.173	14.864	1	<0.001	1.952	1.389	2.742
**Log10(TSH mIU/L) at first trimester of gestation**	0.591	0.128	21.397	1	<0.001	1.806	1.406	2.320
**Positive antithyroid-antibodies**	0.905	0.232	15.233	1	<0.001	2.471	1.569	3.893
**Constant**	−5.629	0.295	364.750	1	<0.001	0.004		

B = coefficient; SE = standard error; df = degrees of freedom; Sig = significance; OR = odds ratio; CI = confidence interval; DM = diabetes mellitus; BMI = body mass index; TSH = thyroid stimulating hormone.

**Table 4:** Multivariate logistic regression analysis including first trimester TSH as a categoric variable (Model 2).

	B	SE	Wald	df	Sig.	Adjusted OR	95% CI by Adjusted OR	
							Lower	Upper
**Age (years)**	0.079	0.008	91.606	1	<0.001	1.082	1.065	1.101
**Family history of type 2 DM**	0.932	0.091	105.646	1	<0.001	2.541	2.127	3.035
**Normal BMI (18.5–24.9 kg/m^2^)**			66.087	3	<0.001			
**Underweight (BMI < 18.5 kg/m^2^)**	0.627	0.265	5.578	1	0.018	1.872	1.113	3.150
**Overweight (BMI 25–29.9 kg/m^2^)**	0.597	0.098	37.217	1	<0.001	1.817	1.500	2.202
**Obesity (BMI ≥ 30 kg/m^2^)**	0.803	0.109	54.560	1	<0.001	2.233	1.804	2.763
**Chronic hypertension**	1.210	0.277	19.024	1	<0.001	3.354	1.947	5.779
**Fetus number (multiple)**	0.658	0.173	14.544	1	<0.001	1.931	1.377	2.709
**TSH between 0.13–2.49 mIU/L**			30.354	3	<0.001			
**TSH < 0.13 mIU/L (Subclinical hyperthyroidism)**	0.190	0.303	0.392	1	0.531	1.209	0.667	2.191
**TSH between 2.5 mIU/L–4.70 mIU/L**	0.429	0.093	21.422	1	<0.001	1.536	1.281	1.842
**TSH > 4.7 mIU/L (Subclinical hypothyroidism)**	0.615	0.158	15.172	1	<0.001	1.849	1.357	2.519
**Thyroid-antibody positive**	0.897	0.231	15.058	1	<0.001	2.453	1.559	3.859
**Constant**	−5.638	0.294	368.382	1	<0.001	0.004		

B = coefficient; SE = standard error; df = degrees of freedom; Sig = significance; OR = odds ratio; CI = confidence interval; DM = diabetes mellitus; BMI = body mass index; TSH = thyroid stimulating hormone.

## Data Availability

The data presented in this study are available on request from the corresponding author.
